# Dawn of the Delphinidans: New Remains of *Kentriodon* from the Lower Miocene of Italy Shed Light on the Early Radiation of the Most Diverse Extant Cetacean Clade

**DOI:** 10.3390/biology13020114

**Published:** 2024-02-11

**Authors:** Francesco Nobile, Alberto Collareta, Vittore Perenzin, Eliana Fornaciari, Luca Giusberti, Giovanni Bianucci

**Affiliations:** 1 Dipartimento di Scienze della Terra, Università di Pisa, Via Santa Maria, 53, 56126 Pisa, Italy; francesco.nobile@phd.unipi.it (F.N.); giovanni.bianucci@unipi.it (G.B.); 2 Dottorato di Ricerca Geoscienze e Ambiente, Università di Pisa, Via Santa Maria, 53, 56126 Pisa, Italy; 3 Museo di Storia Naturale, Università di Pisa, Via Roma 79, 56011 Calci, Italy; 4 Museo Civico Archeologico, Via Lorenzo Luzzo, 23, 32032 Feltre, Italy; sassruisfeltre@gmail.com; 5 Dipartimento di Geoscienze, Università di Padova, Via Giovanni Gradenigo, 6, 35131 Padova, Italy; eliana.fornaciari@unipd.it (E.F.); luca.giusberti@unipd.it (L.G.)

**Keywords:** Bolago Marl, Burdigalian, Delphinida, Friulian-Venetian Basin, Kentriodontidae, narrow-band high-frequency echolocation, Odontoceti, paleobiogeography, paleoecology, proto-Mediterranean

## Abstract

**Simple Summary:**

Nowadays, the infraorder Delphinida consists of oceanic dolphins and porpoises plus a handful of riverine and (sub-)Arctic forms. Overall, the delphinidans account for more than half of the living cetacean species, thus comprising the core of present-day marine mammal diversity. The fossil record indicates that a critical phase of the evolutionary history of Delphinida occurred during the Early Miocene (*c*. 23.0–16.0 million years ago) when the extinct genus *Kentriodon* first appeared and became widespread worldwide. Our paper deals with a new delphinidan fossil from northeastern Italy, namely, an incomplete skull with ear bones dating back to 20.4–19.0 million years ago. This new specimen is recognized herein as a representative of *Kentriodon* and specifically as the first unambiguous member of this genus from the Euro-Mediterranean region. Our new find represents the best candidate for being the most ancient member of *Kentriodon*. The evolutionary success of *Kentriodon* (which lasted until the Late Miocene, less than 11.3 million years ago) may have been favored by the evolution of a peculiar biosonar system exploiting narrow-band high-frequency sounds, which in turn would have been hardly detectable by large-bodied, predatory toothed whales. Furthermore, *Kentriodon* was seemingly characterized by a proportionally larger brain compared to many coeval toothed whales, which in turn may evoke enhanced behavioral capabilities.

**Abstract:**

Nowadays, the infraorder Delphinida (oceanic dolphins and kin) represents the most diverse extant clade of Cetacea, with delphinids alone accounting for more than 40% of the total number of living cetacean species. As for other cetacean groups, the Early Miocene represents a key interval for the evolutionary history of Delphinida, as it was during this time span that the delphinidans became broadly distributed worldwide, first and foremost with the widespread genus *Kentriodon* and closely related forms. Here, we report on a new odontocete find from Burdigalian (20.4–19.0 Ma) deposits of the Friulian-Venetian Basin of northeastern Italy, consisting of the partial cranium of a small delphinidan with associated ear bones (right periotic, stapes, malleus and tympanic bulla). Osteoanatomical considerations and comparisons allow us to assign the studied specimen to the genus *Kentriodon*. This is the first confirmed record of *Kentriodon* from Europe as well as from the whole proto-Mediterranean region. Stratigraphic and phylogenetic considerations suggest that our new specimen may represent the geologically oldest member of *Kentriodon*. The evolutionary success of *Kentriodon* may correlate with the emergence of narrow-band high-frequency echolocation as a possible strategy to escape acoustic detection by large marine predators such as the squalodontids. In addition, the relatively high encephalization quotient of *Kentriodon* spp. may have provided these early dolphins with some kind of competitive advantage over the coeval non-delphinidan odontocetes.

## 1. Introduction

Nowadays, the infraorder Delphinida (Cetacea: Odontoceti) is represented by the so-called river dolphins of the superfamily Inioidea—i.e., *Inia*, *Pontoporia* and the seemingly extinct *Lipotes*, each in its own family—and by the many species that comprise the superfamily Delphinoidea—including the extant oceanic dolphins (family Delphinidae) and porpoises (family Phocoenidae) as well as the narwhal and beluga (family Monodontidae) [1]. Delphinida represents the most diverse extant cetacean clade, with delphinids alone accounting for more than 40% of the total number of living cetacean species [2]. In addition to the many extinct representatives of the aforementioned superfamilies, the fossil record of delphinidans includes a polyphyletic set of more archaic taxa, most of which have often been subsumed into the largely Miocene family Kentriodontidae [3,4,5,6,7]. Overall, the extinct and extant members of Delphinida share a small to intermediate body size, a roughly to fully homodont dentition, a reduced posterior extension of the premaxilla, a posteriorly elongated lateral lamina of the palatine and some peculiar ear bone characters [1,5,8].

Fossils assigned to Delphinida indicate that this clade originated in the late Oligocene, diversified during the Early and Middle Miocene, and then radiated again from the latest Miocene onwards, when the oceanic dolphin family Delphinidae originated and quickly gained ground over other odontocete lineages [1,9,10]. The Aquitanian–Burdigalian interval, in particular, is especially relevant to the fossil history of Delphinida, as it was during this time span that the delphinidans became broadly distributed worldwide, first and foremost with the widespread genus *Kentriodon* and closely related forms [1,9]. 

Here, we report on a new odontocete find from the Lower Miocene deposits of the Friulian-Venetian Basin of northeastern Italy, consisting of the partial cranium of a small delphinidan with associated ear bones (right periotic, stapes, malleus and tympanic bulla). 

Following a thorough characterization of our new find, its paleoecological and paleobiogeographic implications are also discussed with special emphasis on the early evolutionary history of the highly successful infraorder Delphinida.

## 2. Materials and Methods

The anatomical nomenclature follows Mead and Fordyce [11] unless stated otherwise.

Photographs of the cranium were taken using a Nikon D5200 camera, equipped with a Sigma 50 mm f/2.8 macro lens, whereas photographs of the ear bones were taken with a Nikon D850 camera equipped with a Nikon Micro Nikkor AF-S 60 mm f/2.8 G ED macro-lens. Anatomical plates were drawn in Inkscape 1.0.2-2. 

Textured 3D models of the cranium and ear bones were elaborated in the Agisoft Metashape software 1.7.6, masking and aligning 37 photographs for the cranium, 71 for the periotic, 78 for the bulla and 103 for the malleus. These photogrammetric models were scaled in Blender 3.5 to their natural size and can be downloaded from the Supplementary Material File S3.

A smear slide was prepared from the powder extracted from the sedimentary matrix entombing the studied specimen by using a millimeter tungsten carbide spherical drill bit mounted on an electric drill. A calcareous nannofossil assemblage analysis of the smear slide was then performed to determine the geological age of the specimen. The smear slide was examined under a light microscope at 1250× magnification. The presence or absence of the main taxa was checked in a prefixed area of approximately 6–7 mm^2^ (roughly equivalent to 3 vertical traverses; modified after Gardin and Monechi [12]). Following Rio et al. [13], the presence/absence of index species was estimated by quantitative counting relative to a predetermined number of taxonomically related forms (i.e., species of the genus *Helicosphaera* vs. 50 helicoliths; species of the genus *Sphenolithus* vs. 30 sphenoliths).

The institutional abbreviations are as follows: LACM, Natural History Museum of Los Angeles County, Los Angeles, CA, USA; MCAF, Museo Civico Archeologico di Feltre, Feltre, Italy; MGGC, Collezione di Geologia “Museo Giovanni Capellini”, Bologna, Italy; MGP-PD, Museo di Geologia e Paleontologia dell’Università di Padova, Padua, Italy; MUSM, Museo de Historia Natural de la Universidad Nacional Mayor de San Marcos, Lima, Peru; and USNM, National Museum of Natural History, Smithsonian Institution, Washington, DC, USA.

## 3. Geological Framework and Age

Since at least the 17th century, and possibly as early as in Ancient Roman times, the Belluno area (Veneto Region, northeastern Italy) has been home to widespread quarrying activities aimed at extracting sandstone and other hard sedimentary rocks to be manufactured into grindstones [14]. These rocks belong to the so-called “Belluno Molasse”, a thick sedimentary succession that was deposited during the Oligocene and Miocene in the Friulian-Venetian Basin, which at that time was shaped as a peri-Adriatic gulf stretching along the southern margin of the Eastern Alps and receiving abundant terrigenous sediments from the nearby Alpine chain [15,16,17]. Many historic finds of vertebrate fossils are known from localities in the hinterland of Belluno where the Lower Miocene Libàno Sandstone was quarried, including abundant skeletal remains of odontocete cetaceans that were mostly studied and described by Giorgio Dal Piaz in the early 20th century (see Muizon [18] and Del Favero and Fornasiero [19] for historical reviews). These remains account for more than 300 individual specimens, including several holotypes as well as representatives of enigmatic, seemingly endemic lineages such as Dalpiazinidae and Eoplatanistidae [20,21,22,23,24,25,26,27,28,29,30,31,32,33]. Based on paleontological and sedimentological considerations, the odontocete fossils from the surroundings of Belluno are regarded as comprising an estuarine to shallow-marine assemblage [34]. 

**Figure 1:**
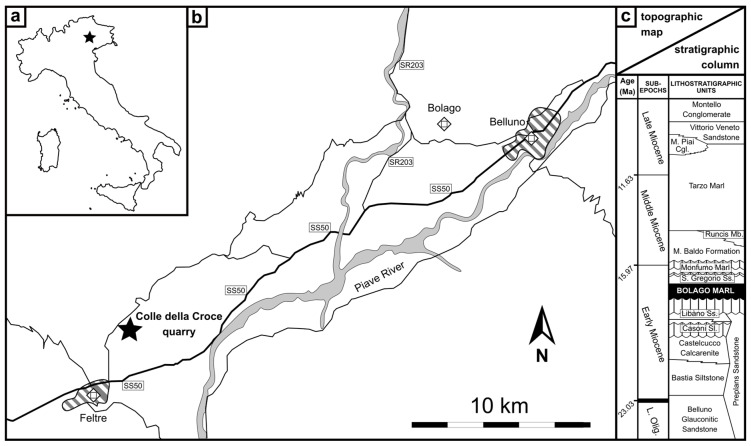
Geographic and stratigraphic setting: (**a**) position of the study area in Italy; (**b**) simplified topographic map of the Belluno area; and (**c**) schematic stratigraphic column thereof, based on a sequence stratigraphy (redrawn and modified from Mellere et al. [17]: figure 3). As for the map, dashed areas indicate the main towns whereas gray areas indicate the main rivers. Our find’s locality (Colle della Croce quarry) as well as the type locality of the Bolago Marl (the eponymous village in the hinterland of Belluno) are also reported in the map. As for the column, the lithostratigraphic provenance of the odontocete fossil described herein is highlighted. Note that our biostratigraphic analyses reveal that the Bolago Marl is slightly geologically older than estimated by Mellere et al. [17]. Abbreviations: Mb. = member; Sl. = siltstone; Ss. = sandstone; SR = Strada Regionale (i.e., regional road); SS = Strada Statale (i.e., state road).

The fossil specimen dealt with in the present paper comes from the Colle della Croce quarry, an as yet largely overlooked fossiliferous locality in the vicinity of Feltre, Belluno Province (Figure 1a,b). Here, quarrying started in the late 1960s to extract a carbonate-rich marl for producing bricks. Shortly after the beginning of the extraction activities, the Colle della Croce quarry became locally famous for hosting abundant shark teeth [35,36] in addition to rarer and typically fragmentary remains of marine mammals [37]. Such fossils occur in deposits assigned to two different formations, namely, the aforementioned Libàno Sandstone (which at Colle della Croce consists of a glauconite-rich basal interval and a fine-grained upper portion) and the overlying Bolago Marl (featuring a highly glauconitic basal horizon that is capped by the aforementioned carbonate-rich marls, which in turn pass upward to glauconite-poor sandstones with finer-grained interbeds) [35,38,39,40]. Our odontocete fossil originates from the early portion of the Bolago Marl, which has been referred to the upper Lower Miocene (Burdigalian) as well as to an outer shelf depositional setting at around 100–200 m water depth in the surroundings of Feltre [17] (Figure 1c).

**Figure 2:**
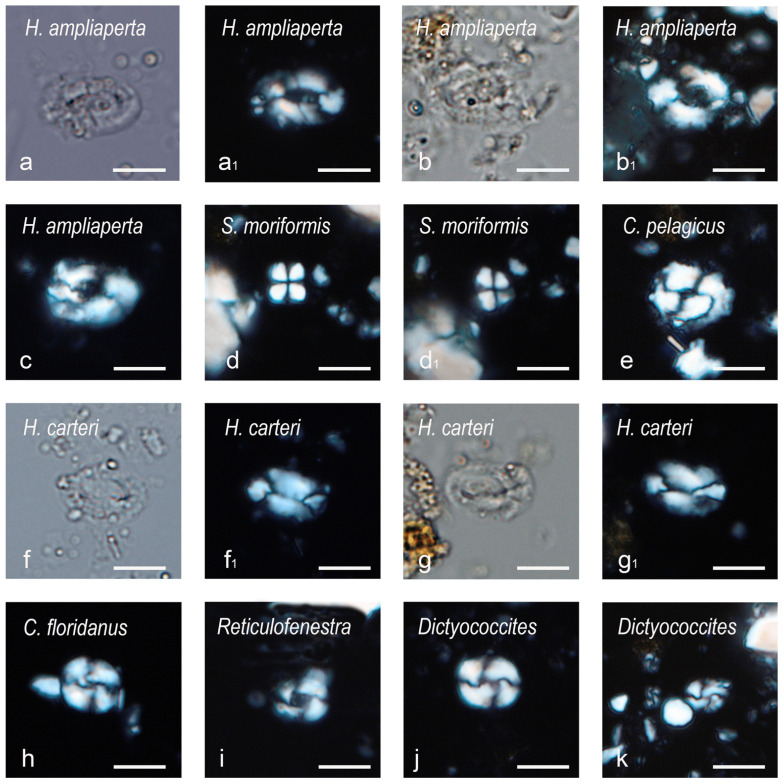
Microphotographs of calcareous nannofossils from the sedimentary matrix entombing the studied specimen. (**a**–**c**) *Helicosphaera ampliaperta* Bramlette and Wilcoxon, 1967 [44]; first specimen (**a**,**a_1_**) in parallel light (**a**) and crossed Nicols (**a_1_**); second specimen (**b**,**b_1_**) in parallel light (**b**) and crossed Nicols (**b_1_**); third specimen (**c**) in crossed Nicols. (**d**,**d_1_**) *Sphenolithus moriformis* (Brönnimann and Stradner, 1960), Bramlette and Wilcoxon, 1967 [44,45], in crossed Nicols 0° (**d**) and crossed Nicols 45° (**d_1_**). (**e**) *Coccolithus pelagicus* (Wallich, 1877), Schiller, 1930 [46,47], in crossed Nicols. (**f**,**g_1_**) *Helicosphaera carteri* (Wallich, 1877), Kamptner, 1954 [46,48]; first specimen (**f**,**f_1_**) in parallel light (**f**) and crossed Nicols (**f_1_**); second specimen (**g**,**g_1_**) in parallel light (**g**) and crossed Nicols (**g_1_**). (**h**) *Cyclicargolithus floridanus* (Roth and Hay, in Hay et al., 1967), Bukry, 1971 [49,50], in crossed Nicols. (**i**) *Reticulofenestra* Hay, Mohler and Wade, 1966 [51], in crossed Nicols. (**j**,**k**) *Dictyococcites* Black, 1967 [52], in crossed Nicols; medium-sized form (**j**); small-sized form (**k**). Scale bars: 5 µm.

Our biostratigraphic analyses of the sedimentary matrix entombing the studied specimen revealed a sparse, moderately well-preserved nannofossil assemblage, consisting mainly of placoliths, the most abundant of which are *Coccolithus*, *Dictyoccites* and *Reticulofenestra*. The genus *Helicosphaera* is common, whereas the genus *Sphenolithus* is scarce but sufficient for statistical counts [41] (see the Supplementary Material File S1 for the raw biostratigraphic data). The presence of common *Helicosphaera ampliaperta* (Figure 2) and the presence of only two questionable specimens of *Sphenolithus* cf. *belemnos* (6.6%) allows the sample to be assigned to the MNN2B and/or the CNM4 biozone of Fornaciari and Rio [41] and Backman et al. [42], respectively, thus constraining its geological age to the early Burdigalian (*sensu* Raffi et al. [43]), corresponding to the ~20.44–19.01 Ma time interval (according to Raffi et al. [43]).

## 4. Systematic Paleontology

Family Kentriodontidae Slijper, 1936 [53] (*sensu* Lambert et al. [54])

**Remarks on the family Kentriodontidae and its taxonomic content**: The systematics of Kentriodontidae are controversial: whereas a few decades ago this family-level name was used to gather most of the extinct genera of Delphinida that fall outside the extant families [5,55], recent phylogenetic analyses have revealed that such a group (“Kentriodontidae *s.l.*”) represents an artificial, polyphyletic set of archaic delphinidans. Therefore, Kentriodontidae has subsequently been redefined (“Kentriodontidae *s.s.*”) and limited in scope to include the type genus *Kentriodon* and a few other closely related genera [6,7,54,56,57,58,59,60]. That said, which and how many genera belong to this family remains controversial. As providing a new contribution to the phylogeny of the *Kentriodon*-like forms is beyond the purposes of the present work, here we follow the phylogenetic approach proposed by Lambert et al. [54], according to which the family Kentriodontidae is restricted to *Kentriodon* and *Rudicetus*. Such a concept of Kentriodontidae has subsequently been confirmed by the phylogenetic reconstructions proposed by Bianucci et al. [59] and Boessenecker and Geisler [60].

**Figure 3:**
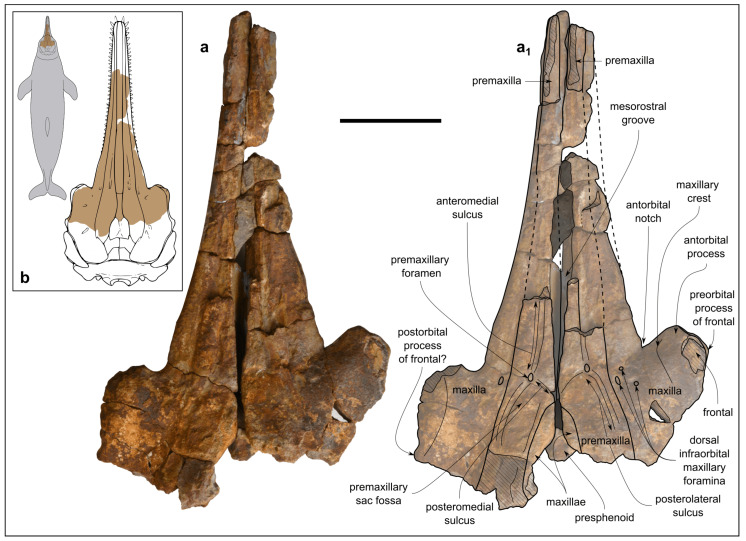
*Kentriodon* sp. (MCAF-MB2), cranium from the Lower Miocene Bolago Marl of northeastern Italy, in dorsal view. Photograph (**a**) and corresponding line drawing (**a_1_**). The grey-shaded areas correspond to hardened sediment. The dashed lines approximate the position of several sutures and borders. Scale bar: 50 mm. (**b**) Anatomical position of the preserved cranium of MCAF-MB2 compared to the dorsal outline of the cranium of *Kentriodon pernix* (right panel, modified from Godfrey and Lambert [61]: figure 2.33) and to the body shape silhouette of a generalized delphinidan (left panel, F.N. own work).

**Figure 4:**
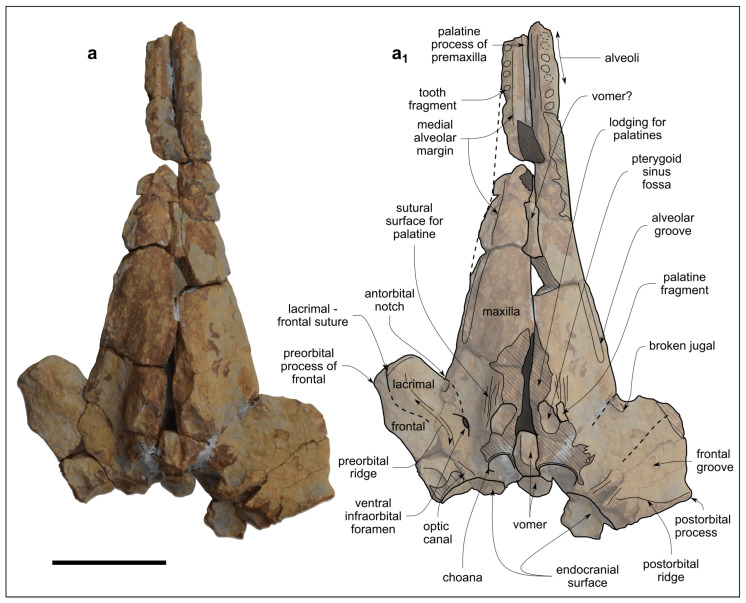
*Kentriodon* sp. (MCAF-MB2), cranium from the Lower Miocene Bolago Marl of northeastern Italy, in ventral view. Photograph (**a**) and corresponding line drawing (**a_1_**). The grey-shaded areas correspond to hardened sediment. The dashed lines approximate the position of several sutures and borders. Scale bar: 50 mm.

**Figure 5:**
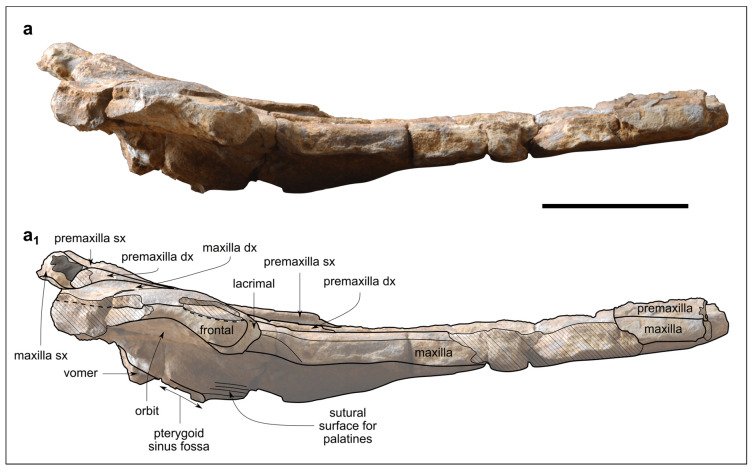
*Kentriodon* sp. (MCAF-MB2), cranium from the Lower Miocene Bolago Marl of northeastern Italy, in right lateral view. Photograph (**a**) and corresponding line drawing (**a_1_**). The grey-shaded areas correspond to hardened sediment. The dashed lines approximate the position of several sutures and borders. Scale bar: 50 mm.

**Figure 6:**
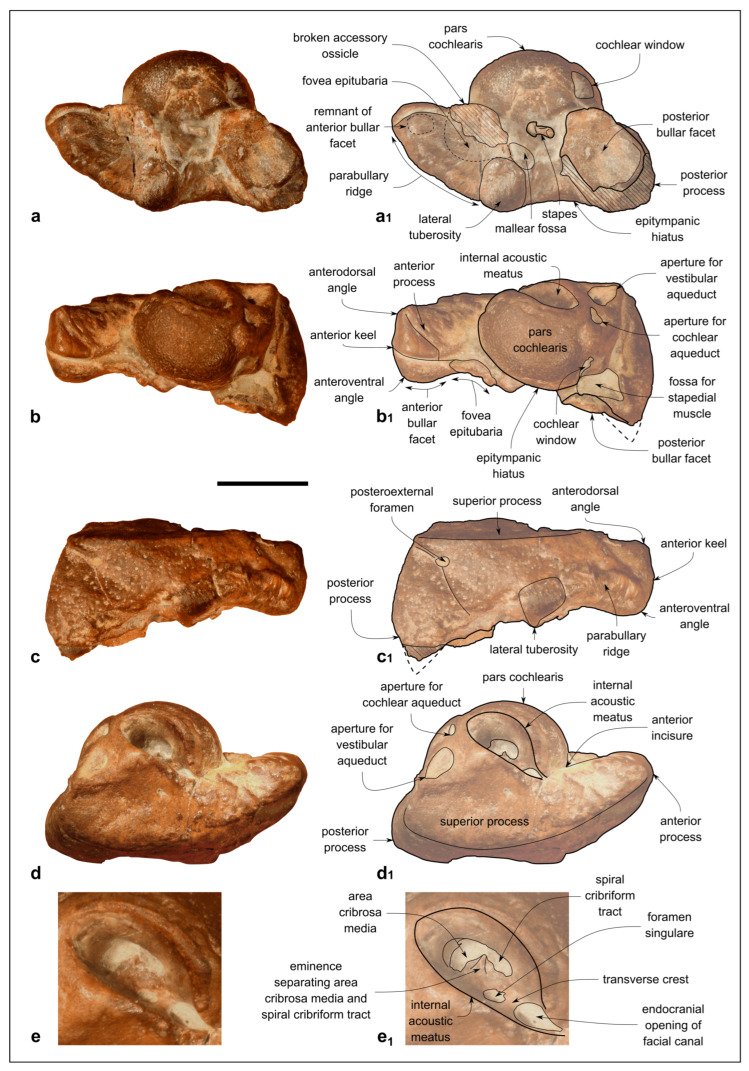
*Kentriodon* sp. (MCAF-MB2), right periotic from the Lower Miocene Bolago Marl of northeastern Italy. (**a**,**a_1_**) Ventral view, photograph (**a**) and corresponding line drawing (**a_1_**); (**b**,**b_1_**) medial view, photograph (**b**) and corresponding line drawing (**b_1_**); (**c**,**c_1_**) medial view, photograph (**c**) and corresponding line drawing (**c_1_**); (**d**,**d_1_**) dorsal view, photograph (**d**) and corresponding line drawing (**d_1_**); (**e**,**e_1_**) close-up of the pars cochlearis in dorsal view, photograph (**e**) and corresponding line drawing (**e_1_**). The dashed lines approximate the position of several sutures and borders. Scale bar: 10 mm (scale bar does not apply to panels **e**,**e_1_**).

**Figure 7:**
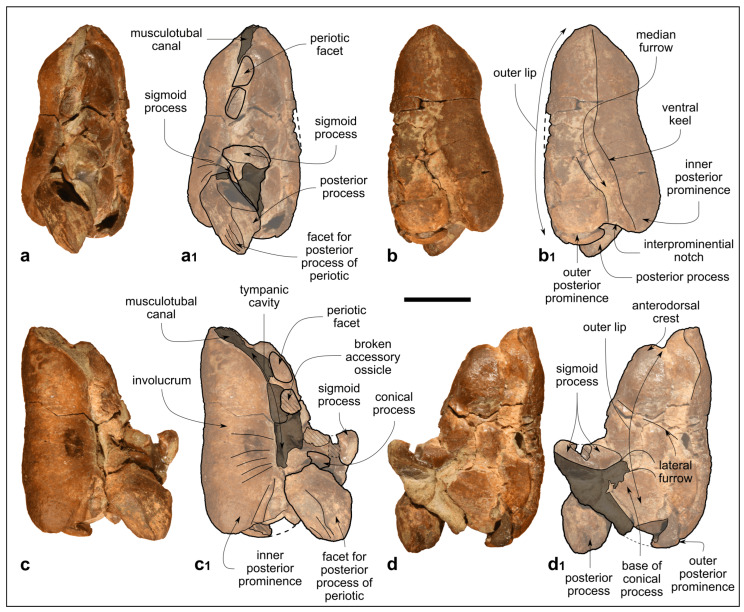
*Kentriodon* sp. (MCAF-MB2), right tympanic bulla from the Lower Miocene Bolago Marl of northeastern Italy. (**a**,**a_1_**) Dorsal view, photograph (**a**) and corresponding line drawing (**a_1_**); (**b**,**b_1_**) ventral view, photograph (**b**) and corresponding line drawing (**b_1_**); (**c**,**c_1_**) medial view, photograph (**c**) and corresponding line drawing (**c_1_**); (**d**,**d_1_**) lateral view, photograph (**d**) and corresponding line drawing (**d_1_**). The grey-shaded areas correspond to hardened sediment. The dashed lines approximate the position of several sutures and borders. Scale bar: 10 mm.

**Figure 8:**
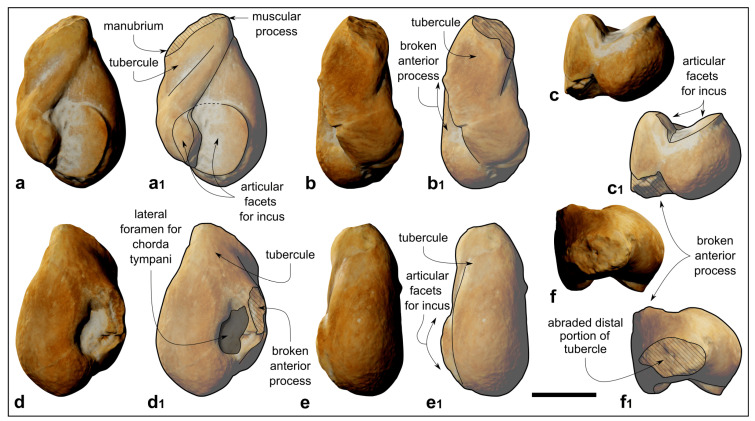
*Kentriodon* sp. (MCAF-MB2), malleus from the Lower Miocene Bolago Marl of northeastern Italy. (**a**,**a_1_**) Posterior view, digital rendering of photogrammetric model (**a**) and corresponding line drawing (**a_1_**); (**b**,**b_1_**) medial view, digital rendering of photogrammetric model (**b**) and corresponding line drawing (**b_1_**); (**c**,**c_1_**) ventral view, digital rendering of photogrammetric model (**c**) and corresponding line drawing (**c_1_**); (**d**,**d_1_**) anterior view, digital rendering of photogrammetric model (**d**) and corresponding line drawing (**d_1_**); (**e**,**e_1_**) lateral view, digital rendering of photogrammetric model (**e**) and corresponding line drawing (**e_1_**); (**f**,**f_1_**) dorsal view, digital rendering of photogrammetric model (**f**) and corresponding line drawing (**f_1_**). The grey-shaded areas correspond to hardened sediment. The dashed lines approximate the position of several sutures and borders. Scale bar: 2 mm.

Genus *Kentriodon* Kellogg, 1927 [3]
*Kentriodon* sp.
(Figure 3, Figure 4, Figure 5, Figure 6, Figure 7 and Figure 8)

**Referred specimen:** MCAF-MB2, an incomplete cranium with an associated tympanic bulla, periotic, stapes and malleus.

**Occurrence:** Northern yard of the Colle della Croce quarry (indicative geographic coordinates: 46°02′51″ N; 11°55′41″ E), near Feltre, Belluno Province, Veneto Region, northeastern Italy. The specimen was found in the Burdigalian Bolago Marl Formation. Standard biostratigraphic analyses of the sedimentary matrix entombing MCAF-MB2 allowed for assigning this specimen to the ~20.44–19.01 Ma time interval.

### 4.1. Description

#### 4.1.1. Overview of the Cranium 

The cranium (Figure 3, Figure 4 and Figure 5) was found broken into several pieces and subsequently reassembled before being studied. Now the fossil appears as a 240-mm-long, incomplete cranium preserving the 77-mm-long anterior portion of the neurocranium and a conspicuous, 163-mm-long part of the rostrum (Figure 3b). The cranium may have undergone some degree of diagenetic deformation, which did not lead to significant changes other than a likely posterior displacement of the left anterior process and slightly different elevations of the dorsal surfaces of the premaxillae at the level of the prenarial triangles. The estimated width of the cranium at the preorbital process is 144 mm. Overall, portions of the premaxillae, maxillae, lacrimals, frontals, palatine, presphenoid and vomer are preserved.

In dorsal and ventral views (Figure 3a,a_1_ and Figure 4), the rostrum is rather transversely wide at the base (79 mm) and gradually tapers anteriorly, reducing to 23 mm at its preserved anterior end. Overall, the lateral margins of the rostrum are weakly concave in dorsal view. The preserved right antorbital notch is V-shaped, with the posterior lateral margin of the rostrum forming an angle of 70 degrees. Although the left antorbital notch is incomplete (the anterior portion of the antorbital process is missing), it is more open than the right antorbital notch. 

In lateral view, the rostrum curves upward anteriorly, while the neurocranium rises slightly posteriorly up to the external bony nares (the posterior most preserved part of the cranium). Overall, the dorsal margin of the cranium is concave in lateral view, drawing a weak arc (Figure 5).

The well-preserved lateral margin of the posterior portion of the rostrum is thick, being about 9 mm high.

#### 4.1.2. Premaxilla 

In dorsal view, the premaxillae are missing for most of the preserved rostrum length, although their lateral margin can be almost entirely traced based on the sutural surface of the underlying maxillae (Figure 3a,a_1_). The lateral margin of each premaxilla shows a weak concavity approximately 15 mm posterior to the right antorbital notch. At the base of the rostrum, the premaxilla is slightly wider transversely than the maxilla. The same proportions seem to be maintained throughout the entire extension of the preserved portion of the rostrum, although it is not possible to establish this with certainty due to the poor preservation state. The medial margins of both the premaxillae are preserved only from *c*. 25 mm anterior to the right antorbital notch where they are 9 mm apart, exposing the mesorostral groove dorsally. The distance between the premaxillae gradually reduces, proceeding posteriorly to a minimum of 3 mm at a level 22 mm posterior to the right antorbital notch. From this point, proceeding posteriorly up to the anterior margin of the external bony nares, the premaxillae diverge, leaving the maxillae exposed dorsally.

A single, small right premaxillary foramen is located 9 mm posterior to the right antorbital notch. The left foramen is smaller than the right one, with a transverse diameter of 2.4 mm compared to 3.4 mm for the right foramen. From each foramen depart well-distinct anteromedial, posteromedial and posterolateral sulci, the last two mentioned margining the anterior portion of the shallow premaxillary fossa.

In ventral view, a transversely narrow portion of premaxillae is exposed in the depressed medial region of the anterior portion of the palatal surface of the rostrum (Figure 4).

In lateral view, the premaxilla rises slightly posteriorly on the neurocranium, reaching a vertical height of *c*. 10 mm from the dorsal margin of the base of the rostrum in correspondence of the external bony nares (Figure 5).

#### 4.1.3. Maxilla 

In dorsal view, the partly preserved rostral portions of the maxillae exhibit a transverse width that remains narrow for most of the length of the rostrum, weakly widening only near the rostrum base. In the better preserved posterior half of the rostrum, each maxilla is almost flat. Posteromedial to the right antorbital notch, a cluster of three small infraorbital foramina pierces the maxilla. The closest to the premaxillary–maxillary suture of these three foramina is also the largest, measuring 2 mm in transverse diameter. Only one left infraorbital foramen is visible, located in the same position and showing the same size as the largest right infraorbital foramen. The line joining these two left and right foramina is slightly posterior to the premaxillary foramina. Lateral to each antorbital notch, one faintly dorsally elevated surface is present, namely, the maxillary crest. The maxillae are well exposed medial to the premaxillae, not only in the aforementioned posterior portion of the mesorostral groove, but also along the entire lateral margin of the partially preserved left external bony naris (Figure 3a,a_1_).

In ventral view, the poorly preserved alveolar rows extend posteriorly up to about 28 mm from the antorbital notches. Along the anterior preserved portion of the rostrum, distinct circular alveoli are observed, ranging in diameter from 3 mm to 4 mm, with interalveolar septa that are *c*. 3 mm thick. The posterior, 60-mm-long portion of the alveolar row is seemingly shaped as a narrow sulcus without distinct alveoli. Although the apparent absence of distinct alveoli could be partly due to the poor state of preservation of the cranium, the posterior narrowing of the alveolar row appears to be a genuine character of the MCAF-MB2 cranium. Posteromedial to the right antorbital notch, the ventral infraorbital foramen is a narrow, 3-mm-long transverse fissure (Figure 4).

#### 4.1.4. Palatine

Only one small fragment of the left palatine is preserved lateral to a concave semicircular depression that represents part of the pterygoid sinus fossa (Figure 4 and Figure 5). Anterolateral to this palatine fragment, the ventral surface of the maxilla shows faint parallel striations that represent the sutural surface for the overlapping, lost portion of the palatine.

#### 4.1.5. Frontal

In dorsal view, the preorbital and supraorbital processes of the frontal are almost completely covered by the maxilla, except for a narrow portion at the lateral margin of the preorbital process and a small, eroded area on the right antorbital process (Figure 3a,a_1_).

In ventral view, the frontal is extensively exposed under the orbit with a faint concave surface named frontal groove, delimited anteromedially by the antorbital ridge and posteriorly by the postorbital ridge. The optic canal is clearly visible at the confluence of these two ridges (Figure 4).

In lateral view, the slender right preorbital process is slightly bent anteroventrally. It articulates with the lacrimal through an arched, anteroventrally convex suture. The supraorbital process of the frontal is thin and has the same vertical height as the covering maxilla. The partially preserved left postorbital process is posteroventrally directed (Figure 5).

#### 4.1.6. Lacrimal and Jugal 

In ventral view, the lacrimal covers the preorbital process of the frontal, although the anteromedial suture with the maxilla and the posterolateral suture with the frontal are barely visible. The preserved maxillary process of the jugal is fused to the lacrimal and located just posteromedial to the antorbital notch (Figure 4). 

In lateral view, the well-preserved right lacrimal marginates the anteroventral border of the antorbital process of the frontal. The preorbital process of the lacrimal has a semicircular, ventrally convex profile (Figure 5).

#### 4.1.7. Presphenoid 

A small piece of the presphenoid (i.e., the anterior part of the nasal septum) takes its place close to the anterior margin of the external bony nares (Figure 3a,a_1_). Otherwise, the presphenoid is not preserved.

#### 4.1.8. Vomer 

Very little is preserved of the vomer, which frustrates any attempt to reconstruct its overall morphology. What remains of this bone is medially exposed in ventral view in a narrow fissure between the maxillae in the middle of the rostrum, as well as with a small piece just anterior to the choanae (Figure 4).

#### 4.1.9. Periotic 

The right periotic is well preserved, only missing a small posterolateral portion of the posterior bullar facet (Figure 6).

The anterior process is 1.16 times as long as the pars cochlearis (measured from its apex to the posterior margin of the lateral tuberosity; see the Supplementary Material File S2 for other measurements in *Kentriodon* and selected basal delphinidan genera) and anteromedially bent, such that its medial margin forms an angle of 70° with the anterior margin of the pars cochlearis (i.e., the anterior incisure; Figure 6a–d_1_). The transverse section of the anterior process is weakly compressed medioventrally at mid-length. In medial and lateral views, the anterior process is roughly rectangular in shape. Its dorsal margin forms a straight line with the dorsal margin of the superior process, whereas its ventral margin is faintly concave. The anterodorsal angle is square and about 90°, whereas the anteroventral angle is rounded. Two sulci are observed on the medial surface of the anterior process. The deeper sulcus runs anteroposteriorly from the midpoint of the anterior keel to the anterior margin of the pars cochlearis. The second sulcus (possibly the anterointernal sulcus of Fordyce [62]) is a weak groove that crosses the medial surface of the anterior process obliquely, starting from the anterodorsal angle (Figure 6b,b_1_). In ventral view, between the apex of the anterior process and the fovea epitubaria, a small vestigial articular surface for the tympanic bulla is observed. Indeed, by articulating the periotic with the tympanic bulla, this surface is revealed to fit in a deep elliptical pit on the dorsal surface of the outer lip (i.e., the periotic facet *sensu* Fordyce [62]). Posterior to the vestigial articular surface for the tympanic bulla, the wide, oval fovea epitubaria is partially obscured by a fragment of the accessory ossicle of the tympanic bulla, still fused with the anterior process of the periotic. Posterior to the fovea epitubaria, a small mallear fossa opens posteromedially. The lateral margin of the anterior process is delimited by the parabullary ridge, which extends from the anteroventral corner to the lateral tuberosity.

The low, hemispherical pars cochlearis displays a deep, drop-shaped internal acoustic meatus on its dorsal surface (Figure 6d–e_1_). The latter exhibits a narrow incisure due to the anterolateral position of the endocranial opening of the facial canal. Within the internal acoustic meatus, the foramen singulare is equally distant from the spiral cribriform tract and the endocranial opening of the facial canal (Figure 6e,e_1_). On the posterodorsal part of the pars cochlearis, the apertures for the cochlear aqueduct and vestibular aqueduct are closer to the posterior margin of the pars cochlearis than to the posterior edge of the internal acoustic meatus. The posterolateral rim of the aperture for the vestibular aqueduct is dorsomedially elevated. Ventral to the aperture for the cochlear aqueduct, the circular cochlear window is closer to the aperture for the vestibular aqueduct than to the cochlear window. 

Lateral to the pars cochlearis, in dorsal view, a wide flat surface (superior process *sensu* Kasuya [63]) is delimited by a prominent, curved lateral ridge (Figure 6c–d_1_).

Lateral to the pars cochlearis, in ventral view, the stapes is still articulated to the fenestra ovalis, and the stapedial muscle fossa is almost totally obscured by the facial crest of the periotic (*sensu* Mead and Fordyce [11]). Lateral to the stapes, the epitympanic hiatus is anteroposteriorly wide. 

The short posterior process bends posterolaterally to form an angle of about 90° with the flat superior process, as clearly visible in medial and lateral views (Figure 6b–c_1_). In ventral view, the posterior bullar facet is smooth and concave, its main axis being directed posterolaterally. A small postero-external foramen is visible on the lateral surface of the posterior process. 

#### 4.1.10. Tympanic Bulla 

The right tympanic bulla is almost complete and moderately well preserved, except for the broken accessory ossicle and the highly fractured lateral wall of the outer lip (Figure 7). 

In ventral view, the tympanic bulla is rather narrow, although this character could have been accentuated by diagenetic compression. The anterior margin forms an angle of *c*. 95°, without an anterior spine. The medial margin is sigmoid due to the anterior expansion of the involucrum, whereas the lateral margin is gently rounded (but this margin is deformed by the crushing of the outer lip). The outer posterior prominence is slightly wider and more posteriorly extended than the inner posterior prominence. These prominences are separated from each other by a deep interprominential notch, which is followed anteriorly by a marked medial furrow. A sigmoid ventral keel runs from the posteromedial margin of the interprominential notch to the apex of the tympanic bulla (Figure 7b,b_1_).

In medial view, the involucrum displays a weakly concave ventral margin and a sigmoid dorsal margin without incisure. The posteromedial angle is *c*. 70°. The dorsomedial surface of the involucrum shows several shallow transverse creases. A deep elliptical fossa, representing the periotic fossa, lies on the anterodorsal crest of the outer lip, followed posteriorly by the broken accessory ossicle. The apex of the conical process is medially visible in the narrow space between the sigmoid process and the posterior process. The posterior process is dorsolaterally bent and displays a convex and elongated facet for receiving the posterior process of the periotic (Figure 7c,c_1_). 

In lateral view, the dorsal margin of the outer lip features an anterior notch due to an abrupt widening of the outer lip at the level of the anterodorsal crest. The ventral margin of the tympanic bulla is weakly concave, and the posterolateral angle is rounded. An oblique, elongated lateral sulcus is still visible along the collapsed lateral surface of the outer lip (Figure 7d,d_1_). The high sigmoid process projects orthogonal to the main axis of the tympanic bulla. 

In anterior view, the musculotubal canal displays an oval section with a dorsoventral diameter of 9.6 mm and a mediolateral diameter of 1.8 mm. 

In posterior view, a small elliptical foramen is visible ventral to the posterior process.

#### 4.1.11. Malleus 

The right malleus lacks the anterior process and exhibits an abraded distal part of the tubercule (Figure 8).

The overall shape of the malleus is pyriform in posterior view due to a mediolaterally expanded head of the malleus (which comprises the ventral half thereof) as well as to an elongated tubercule (Figure 8a–d_1_). The medial articular facet for the incus forms a 90° angle with the smaller lateral facets (Figure 8c,c_1_,f,f_1_). The muscular process is placed distinctly more dorsally than the manubrium (Figure 8a,a_1_).

#### 4.1.12. Stapes 

The stapes is still articulated with the periotic and partly embedded in hard cemented sediment (Figure 6a,a_1_). The slender, fused anterior and posterior crura and the small head of the stapes are visible.

### 4.2. Comparisons 

**Figure 9:**
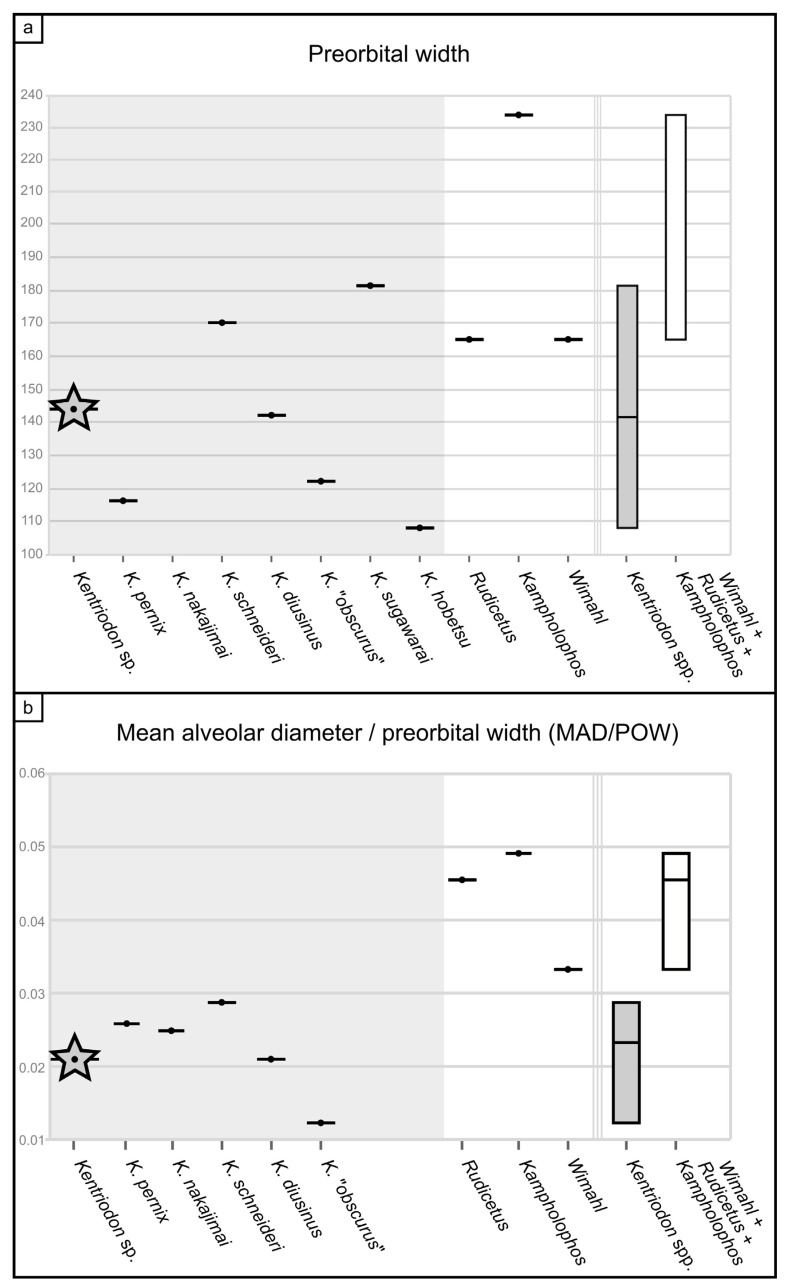
Morphometric comparisons. (**a**) Preorbital width (in mm) of *Kentriodon* sp. (=MCAF-MB2, indicated by a star), other *Kentriodon* spp. and other basal delphinidan taxa; on the right, boxplots indicate the range of intrageneric variability in *Kentriodon* compared to other basal delphinidans. (**b**) Ratio between the mean alveolar diameter and the preorbital width (both measurements in mm) in *Kentriodon* sp. (=MCAF-MB2, indicated by a star), other *Kentriodon* spp. and other basal delphinidan taxa; on the right, boxplots indicate the range of intrageneric variability in *Kentriodon* compared to other basal delphinidans (note that the two boxplots do not overlap with each other). See the Supplementary Material File S2 for data sources.

Overall, the fragmentary cranium of MCAF-MB2 mostly resembles *Kentriodon* in terms of its small size, gracile appearance and general proportions (e.g., the rostrum being wide and dorsally flat at its base and abruptly tapering anteriorly). Compared to other roughly coeval delphinidans that are closely related to *Kentriodon* (i.e., *Rudicetus* Bianucci, 2001 [64], *Kampholophos* Rensberger, 1969 [65] and *Wimahl* Peredo, Uhen and Nelson, 2018 [6]), the cranium of MCAF-MB2 displays a smaller preorbital width (a rough proxy of cranial size; Figure 9a), as is also the case for most *Kentriodon* species, whereas its dental alveoli are proportionally smaller, as in all *Kentriodon* species (Figure 9b). *Brevirostrodelphis* Godfrey and Lambert, 2023 [61], another basal delphinidan that in some phylogenies is recovered as a close relative of *Kentriodon* (e.g., Lambert et al. [66]), is similar to MCAF-MB2 in terms of cranial and alveolar size but differs significantly from our new specimen by displaying a shorter and more tapered rostrum. Therefore, our comparisons are restricted to the members of the genus *Kentriodon* and especially to the following species that are based on diagnostic cranial materials: *Kentriodon diusinus* Salinas-Márquez et al., 2014 [67], *Kentriodon hobetsu* Ichishima, 1994 [68], *Kentriodon nakajimai* Kimura and Hasegawa, 2019 [58]; *Kentriodon obscurus* Barnes and Mitchell, 1984 [69], *Kentriodon pernix* Kellogg, 1927 [3], *Kentriodon schneideri* Whitmore and Kaltenbach, 2008 [70] and *Kentriodon sugawarai* Guo and Kohno, 2021 [71]. In turn, other nominal species of *Kentriodon* (e.g., *Kentriodon fuchsii* (Brandt, 1873) [72] and *Kentriodon hoepfneri* (Kazár and Hampe, 2014) [73]) are not taken into account herein because they are based on undiagnostic material and/or their assignment to the genus *Kentriodon* is tenuously supported in our opinion. In particular, *K. fuchsii* has a troubled taxonomic history: first described as a new species of the genus *Champsodelphis* based on a heterogenous set of specimens that included the type materials of *Phocaena* [sic] *euxinica* Nordmann, 1860 [74] and *Delphinus fossilis bessarabicus* Nordmann, 1860 [74], as well as new postcrania from the upper Middle Miocene (Sarmatian) of Nussdorf (Vienna, Austria), its validity was questioned as early as by Van Beneden and Gervais [75]. Subsequently, Pia [76] limited the hypodigm of Brandt’s species (which he treated under the combination *Acrodelphis fuchsii*) to the material from Nussdorf. Following in Pia’s footsteps, Kazár et al. [77] regarded the type of *C. fuchsii* as only comprising postcrania, which however are largely undiagnostic for odontocetes in general [78] and the oceanic dolphins’ ancestors in particular [79]. This implies that Kazár et al.’s [77] attribution of several ear bones (periotics and bullae, including a tympano–periotic complex associated with a partial skeleton) to Brandt’s species (which they treated under the combination *Atocetus* (?) *fuchsii*) is arbitrary because the type material of the latter features no ear bones nor any other cranial remains. As a consequence of this, Kazár’s [80] attribution of *C. fuchsii* to the genus *Kentriodon* (which is based on similarities between many isolated periotics from the Middle Miocene of the Vienna Basin and the corresponding bones of *K. pernix* and *K. obscurus*) is also questionable. With respect to *K. hoepfneri*, its cranium is too fragmentarily known for warranting an attribution to the genus *Kentriodon*, whereas its periotic exhibits marked pontoporiid affinities such as an overall globular shape due to the very short anterior and posterior processes, a thick dorsal process and an incipient, flat, thin posterior expansion of the posterior process. 

A different issue concerns *K. obscurus*, whose holotype is an isolated periotic that was originally described as *Grypolithax obscura* by Kellogg [81]. The combination *Kentriodon obscurus* was proposed by Barnes and Mitchell [69] who arbitrarily attributed an isolated fragmentary cranium without ear bones (LACM 21256) to Kellogg’s species. Barnes and Mitchell [69] also assigned to *K. obscurus* several isolated periotics from the same bonebeds as the LACM 21256 cranium; however, this set of periotics displays a rather high degree of morphological variability that may be suggestive of interspecific differences. Therefore, since the LACM 21256 cranium and the *Grypolithax obscura* holotype periotic may belong to different species, here we limit our comparisons to the former, which we refer to as representative of *Kentriodon* “*obscurus*”. 

In this context, it is also worth mentioning that the Belluno Molasse is home to another putative kentriodontid in addition to MCAF-MB2, namely, *Protodelphinus capellini* Dal Piaz, 1977 [33]. Coming from the Libàno Sandstone, *P. capellini* is based on a fragmentary rostrum, incomplete mandibles, right ear bones and some teeth, all of which belong to the holotype and only known specimen (MGP-PD 26182–26186). According to Dal Piaz [33], *P. capellini* represents a basal Delphinidae, whereas Muizon [82] referred this taxon to the family Eurhinodelphinidae based on ear bone characters. Lambert [83] pointed out that the mandibles of *P. capellini* lack some of the typical characters of the eurhinodelphinids (e.g., a longitudinal groove on the lateral bone surface) and may rather belong to a kentriodontid. Therefore, Lambert [83] questioned whether the mandibles belong to the same cetacean individual as the associated, eurhinodelphinid-like ear bones. However, subsequent research has shown that no conspicuous lateral groove occurs in an indeterminate eurhinodelphinid from the Lower Miocene of Peru [84]. Therefore, considering also the absence of unambiguous kentriodontid (or even delphinidan) characters in the mandibles of *P. capellini*, we contend that the most parsimonious hypothesis is that MGP-PD 26182–26186 represents a single odontocete taxon and specimen close to the basal eurhinodelphinids. 

#### 4.2.1. Cranium 

The cranium of MCAF-MB2 displays remarkable affinities with that of *Kentridon pernix*, the type species of the genus, by having a V-shaped right antorbital notch, premaxillary and dorsal infraorbital foramina that are located slightly posterior to the rostrum base, a low maxillary crest, a thin roof of the orbit and a concave orbital margin of the supraorbital process of frontal [3,61]. However, the cranium of MCAF-MB2 differs from that of *K. pernix* by its larger size, smaller ventral infraorbital foramen, more robust posterolateral margin of the rostrum and smaller dental alveoli (Figure 9). With respect to the last three characters, MCAF-MB2 is more reminiscent of *Kentriodon “obscurus”*, which however displays narrower antorbital notches and a medially bent posterior end of the alveolar row (Barnes and Mitchell, 1984) [69]. Moreover, MCAF-MB2 also differs from both *K. pernix* and *K. “obscurus”* in that most of the preorbital process and supraorbital process of the frontal are covered by the maxilla dorsally; in addition, the anterior margins of the pterygoid sinus fossae, which are apparently well-separated medially, are rounded rather than triangular and are not extended anteriorly beyond the level of the antorbital notches. Besides MCAF-MB2, these characters are also present in *Kentriodon hobetsu*, *K. schneideri* and *K. sugawarai* but not in *K. diusinus* and *K. nakajimai*. *Kentriodon hobetsu* further shares with MCAF-MB2 a wide medial exposure of the maxilla along the margin of the external bony nares, which is absent in all other species of *Kentriodon* that have been formally described to date [68]. On the other hand, *K. hobetsu* differs from MCAF-MB2 and all other species of *Kentriodon* by its smaller size (possibly due to the young ontogenetic age of the holotype and only known specimen) as well as by a marked constriction of the premaxillae at the base of the rostrum, the lateral margin of each premaxilla being strongly concave in dorsal view.

*Kentriodon schneideri* differs from MCAF-MB2 by being larger (possibly due to the old age of the holotype and only known specimen) as well as by exhibiting a weaker constriction of the dorsal opening of the mesorostral groove around the rostrum base, more anteriorly located premaxillary foramina, wider and U-shaped antorbital notches, and proportionally larger dental alveoli [70]. 

The cranium of *K. sugawarai* further differs from that of MCAF-MB2 by its larger size as well as in having an unusually small antorbital process, a consequently low antorbital notch, more anteriorly placed premaxillary and anterior dorsal infraorbital foramina, and a thinner roof of the orbit [71]. 

*Kentriodon diusinus* further differs from MCAF-MB2 by featuring a transversely narrower preorbital process and antorbital notch, and a more open mesorostral groove around the rostrum base [67]. 

The cranium of *K. nakajimai* further differs from that of MCAF-MB2 by its more abruptly tapering rostrum, more salient maxillary crest, narrower antorbital notch, rounded anterior margin of the antorbital notch, dorsoventrally thinner supraorbital process, and wide and well-distinct fossa for the preorbital lobe of the pterygoid sinus [58]. 

#### 4.2.2. Periotic 

The periotic of MCAF-MB2 exhibits the typical features observed in the genus *Kentriodon* as well as in other basal members of Delphinida, namely: anterior and posterior processes that are inclined anteromedially and posterolaterally, respectively, hence the overall sigmoidal shape of the periotic in dorsal and ventral views; an apex of the anterior process that is squared in lateral and medial views (a character shared with all delphinidans); a wide, elliptical, shallow fovea epitubaria; the lack of an evident anterior bullar facet; a ventrolaterally bent posterior process; and a bullar facet without deep ridges. Moreover, the periotic of MCAF-MB2 has a flat superior process delimited by a prominent, curved lateral ridge (a feature that occurs in the periotics of all *Kentriodon* species but *K. nakajimai*).

Compared to the periotic of the holotype of *K. pernix*, the periotic of MCAF-MB2 exhibits an anterior process that is more elongated and more pointed in dorsal and ventral views. This significant difference is due to the fact that MCAF-MB2 retains a vestigial anterior facet anterior to the fovea epitubaria, which is absent (or at least more reduced) in *K. pernix.* On the other hand, the fovea epitubaria of MCAF-MB2 is less expanded laterally than that of *K. pernix*. MCAF-MB2 also differs from *K. pernix* by displaying a shallower hiatus epitympanicus, an elliptical rather than circular internal acoustic meatus and a ventral edge of the anterior process that is concave in lateral view [3,61]. 

The periotic of the holotype of *K. sugawarai* is very similar to that of MCAF-MB2, from which it differs by displaying an unusually wide and low epitympanic hiatus in ventral view (e.g., Guo and Kohno [71]: figure 8A, which however may be slightly oriented ventrolaterally). Interestingly, both the periotic of *K. sugawarai* and that of MCAF-MB2 feature a flat vestigial anterior facet. 

The periotic referred to *K. nakajimai* clearly differs from that of MCAF-MB2 by lacking a distinct, flat superior process, as well as by the shorter and pointed anterior process, less mediolaterally compressed pars cochlearis and circular internal acoustic meatus [58].

#### 4.2.3. Tympanic Bulla 

The tympanic bulla of MCAF-MB2 features a posterodorsal excavation of the involucrum as observed in all delphinidans [5]. Moreover, the involucrum of MCAF-MB2 exhibits an anterior expansion that forms a faintly concave medial margin (in ventral view) and a faintly concave ventral margin (in medial view), as also observed in many other delphinidans.

The tympanic bulla of the holotype of *K. pernix* is close to that of MCAF-MB2 in several characteristics, including the deep median furrow, the elongated and sigmoid ventral keel, the absence of an anterior spine, the elevated sigmoid process and the elongated lateral furrow. However, *K. pernix* differs from MCAF-MB2 by displaying a straight medial margin in ventral view, a posterior process that projects posteroventrally rather than posterodorsally and a smaller anterodorsal crest of the outer lip devoid of the peculiar elliptical periotic facet [3].

The incomplete tympanic bulla of the holotype of *K. sugawarai* is overall consistent with that of MCAF-MB2 even if it seemingly lacks the ventral keel [71]. 

The tympanic bulla referred to *K. nakajimai* is deformed and badly preserved, thus frustrating any accurate comparison with MCAF-MB2. That said, judging from Kimura and Hasegawa’s [58] figure 6, the former does not significantly differ from the latter, except perhaps for a more anteriorly extended median furrow and a weaker lateral furrow.

#### 4.2.4. Malleus 

The malleus of MCAF-MB2 exhibits a well-developed muscular process that is located distinctly more dorsally than the manubrium, a synapomorphy of Delphinida according to Muizon [5]. Although this character is not always evident in delphinidans (e.g., Bianucci et al. [85]), its presence in MCAF-MB2 further supports the assignment of this specimen to Delphinida.

**Figure 10:**
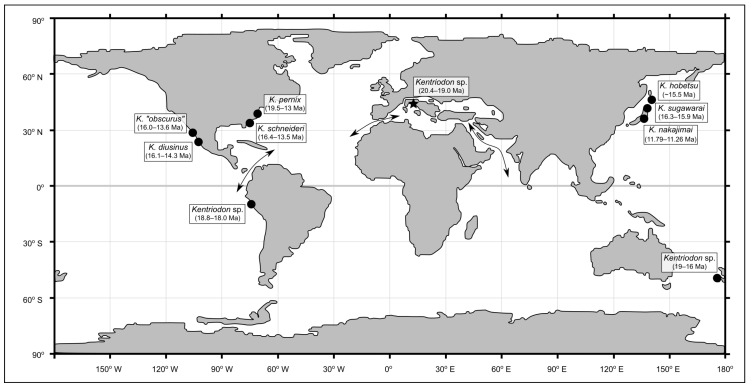
Paleogeographic map of the Early Miocene, redrawn from He et al. ([105]: figure 4), showing the distribution of the unambiguous records of the genus *Kentriodon*. The chronostratigraphic intervals derive from several previous publications [7,61,95,97] and the present work. Arrows indicate the paleogeographic routes that were open during the Miocene (Early Miocene only for the Eastern Tethys Seaway), linking the proto-Mediterranean, the Atlantic Ocean, the Indian Ocean and the Pacific Ocean.

**Figure 11:**
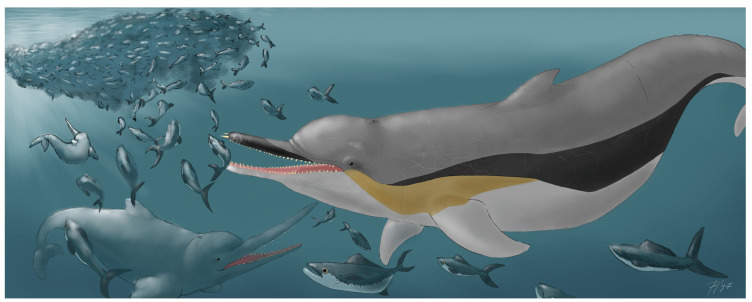
Paleoartistic reconstruction of *Kentriodon* sp. in the Early Miocene Belluno Molasse paleoenvironment. The body proportions derive from Kellogg ([3]: figure 5) and Godfrey and Lambert ([61]: figure 2.33). The associated fauna includes bony fish and toothed whales (family Eurhinodelphinidae), both of which were mentioned by Dal Piaz [32]. Artwork by F.N.

The malleus of the holotype of *K. pernix* ([3]: figures 8–13) clearly differs from that of MCAF-MB2 by displaying a smaller tubercule (ratio between the length of the tubercule and the total length of the malleus, Lt/Lm = 0.37 in *K. pernix*, *contra* 0.52 in MCAF-MB2) and an unusually short processus muscularis (slightly less developed than the manubrium). 

The processus muscularis of the malleus of the holotype of *K. sugawarai* [71] is also low and less developed than the manubrium, whereas its tubercle is narrower and more elongated than observed in MCAF-MB2 (Lt/Lm = 0.54).

#### 4.2.5. Concluding Comparison Remarks 

The above comparisons indicate that MCAF-MB2 possesses a mosaic of characters that are displayed by various species of *Kentriodon*. Furthermore, the presence of a peculiar articulation between the anterior bullar facet and the periotic facet may represent a distinctive character of this extinct dolphin form. These considerations suggest that MCAF-MB2 may belong to an as yet undescribed species of *Kentriodon*. However, considering the incompleteness of some diagnostic parts of the skull (e.g., the vertex), we conservatively identify MCAF-MB2 as belonging to *Kentriodon* sp.

## 5. Discussion and Conclusions

The fossil specimen described herein provides the first unambiguous record of *Kentriodon* from Europe as well as from the whole proto-Mediterranean region (the Eastern Tethys Seaway was still open in early Burdigalian times, so a true Mediterranean Basin still had to form [86,87]). As such, it further highlights the wide geographic distribution of this basal delphinidan genus. Indeed, by considering the occurrence data that are based on diagnostic cranial materials, *Kentriodon* is revealed as one of the fossil cetacean genera with the widest geographical distribution (Figure 10). The reasons behind such a quasi-cosmopolitan distribution may be linked to the likely pelagic habitat preferences of this small cetacean, which may have been reminiscent of the similarly sized, extant delphinid genera *Delphinus* and *Stenella* in this regard (see Fordyce and Barnes [88] and Ichishima et al. [89] for a similar hypothesis) (Figure 11). These considerations further suggest that the dispersal capabilities of *Kentriodon* were distinctly higher than those of many other common forms of Early to Middle Miocene odontocetes, including *Squalodon*, which in turn was likely used to more nearshore and even brackish settings (as also evoked by the high abundance of fossils referred to this genus in the deltaic to shallow-marine deposits of the Libàno Sandstone) [90]. Additional analyses of the vertebral column of *Kentriodon* may clarify whether its postcranial anatomy was particularly well-suited for an efficient swimming style compared to other coeval odontocetes (but see also Gillet et al. [91]).

When considering the fossil record of *Kentriodon* spp. from a stratigraphic point of view, only *K. pernix* from the Calvert Formation of Maryland (USA) overlaps in terms of estimated geological age with MCAF-MB2, whereas all other members of *Kentriodon* appear to be younger (Figure 10). The broad geochronological range (19.5–13.0 Ma) of *K. pernix* reflects the large sample of fossils referred to this species from many different strata of the Calvert Cliffs [61]. In particular, the geologically oldest specimens of *K. pernix* originate from the Fairhaven Member, whose estimated age (19.5–19.0 Ma [61]) partially overlaps with that of the MCAF-MB2 specimen (20.4–19.0 Ma; this work). Therefore, as early as 19.5–19.0 Ma, *Kentriodon* was already present in the eastern North Atlantic as well as in the proto-Mediterranean region. That said, the specimen from the Colle della Croce quarry represents the best candidate for being the most ancient member of *Kentriodon*, not only in light of its maximum biostratigraphic age (which at 20.4 Ma approximates the Aquitanian–Burdigalian boundary as conceived at present) but also because other lines of evidence suggest that this basal delphinidan originated in the proto-Mediterranean region. First and foremost, most phylogenetic reconstructions recover *Kentriodon* as the sister group of *Rudicetus* from the Lower Miocene of southern Italy [64]. In addition, several isolated *Kentriodon*-like ear bones are known from the Burdigalian deposits of the proto-Mediterranean area as well as from the nearby Paratethys, including the periotics referred to cf. *Kentriodon* from the Upper Marine Molasse (21–17 Ma) of Switzerland [92] and the ear bones referred to Kentriodontidae indet. from the Pietra da Cantoni Group (19–16 Ma) of northwestern Italy [93].

Regardless of whether *Kentriodon* originated in the Mediterranean and then rapidly dispersed into the North Atlantic or vice versa, its subsequent dispersal likely occurred through the Central American Seaway and along the western coasts of the South Pacific. In fact, in the well-studied Chilcatay Formation (19.3–18 Ma) of Peru, *Kentriodon* suddenly appears with several specimens just above a volcanic ash layer dated at 18.85–18.15 Ma, thus becoming the dominant form of the whole Chilcatay assemblage of fossil cetaceans [94,95,96]. With respect to the southern Pacific realm, two undescribed *Kentriodon* skulls phenetically close to *K. pernix* are also known from the Burdigalian Caversham Sandstone of New Zealand [97]. However, the precise position of these fossils along the temporally long (about 19–16 Ma) stratigraphic succession of the Caversham Sandstone is not known, which means that any paleobiogeographic interpretation of the New Zealand record of *Kentriodon* would be speculative at present. In turn, more robust data support the arrival of *Kentriodon* in the North Pacific around 16 Ma ago, both along the Eastern Pacific coast, with *K. diusinus* (16.1–14.3 Ma) and *K.* “*obscurus*” (16–13.6 Ma) [67,69], and along the western Pacific coast, with *K. sugawarai* (16.3–15.9 Ma), *K. hobetsu* (*c*. 15.4 Ma) and *K. nakajimai* (11.79–11.26) [58,68,71]. *Kentriodon nakajimai* appears to be the last surviving species of *Kentriodon*, suggesting its disappearance during the Late Miocene, less than 11.3 Ma [58].

It should be noted that the above paleobiogeographic discussion has taken into account the spatial and temporal distribution of the fossil record of *Kentriodon* but not the phylogenetic relationships among the various members of this genus. Indeed, as already mentioned, although several recent cladistic analyses have investigated the basal delphinidans, relationships remain volatile for “Kentriodontidae *s.l.*” as well as for “Kentriodontidae *s.s.*”, thus frustrating any attempt to address the paleobiogeographic history of *Kentriodon* in a more rigorous way. For example, Kimura and Hasegawa [58] built upon their phylogenetic analysis (which recovers *K. diusinus* as the basalmost *Kentriodon* species) to suggest that *Kentriodon* originated in the Pacific Ocean and then repeatedly migrated into the Atlantic Ocean. On the other hand, Guo and Kohno [7] regarded *K. schneideri* from the North Atlantic as the earliest branching *Kentriodon* species. These discrepancies are also due to the fragmentary nature of the fossil record, as many *Kentriodon* species are only known by a single specimen and/or from a single fossiliferous locality.

The fossil history of *Kentriodon* is clearly one of a globally widespread, long-lasting genus, but the reasons behind such a success remain by large obscure. Interestingly, a recent study indicates that at least one member of *Kentriodon* (namely, *K. pernix*) may have been among the earliest toothed whales to produce narrow-band high-frequency (NBHF) sounds based on cochlear dimensions. NBHF echolocation is currently used by several small-sized odontocetes (mostly delphinidan species) as a possible strategy to escape acoustic detection by large marine predators such as killer whales [98]. Thus, the predatory pressure of *Squalodon* (a possible analog of the extant orca [1,99]) may have favored the emergence of this peculiar ability in *Kentriodon*, thus preluding to the key role of NBHF adaptations in the subsequent radiation of the small delphinoids (see Aguirre Fernandez et al. [92] for a similar hypothesis). As a matter of fact, *Squalodon* is common in the Libàno Sandstone as well as in the oldest strata of the Calvert Formation (note that the giant species *Squalodon whithmorei* reached 5.5 m in total body length [100]). 

The relatively high degree of encephalization of *Kentriodon* spp. may also be relevant here. Based on quantitative data on brain and body size for 36 extinct cetacean species, Marino et al. [101] recognized that brain size increased significantly during two critical phases of the odontocete evolution, namely, with the origin of Odontoceti from the ancestral group Archaeoceti around the Eocene–Oligocene transition and with the Miocene emergence of the delphinidans (see also Boessenecker et al. [102] and Bisconti et al. [103]). Marino et al. [101] reported encephalization data for two species of *Kentriodon*, namely, *K. pernix* and *K. schneideri*, which appear to display brains that are proportionally larger than those of most squalodontids, eurhinodelphinids and physeteroids, and roughly comparable with those of the extant porpoises. Thus, it is tempting to speculate that the relatively large brains of *Kentriodon* spp. provided these early dolphins with some kind of competitive advantage over the coeval non-delphinidan odontocetes. For example, although a formal analysis of the link between social group size and brain size is still wanting [104], *Kentriodon* has sometimes been reconstructed as a social odontocete that used to form large schools, thus resembling the extant oceanic dolphins in this respect [89].

The hopeful discovery of more complete specimens of *Kentriodon* from the Colle della Croce quarry may prove pivotal for further elucidating the reasons behind the success of this widespread, speciose, long-lasting genus.

## Data Availability

The data presented in this study are available in Supplementary Materials.

## Supplementary Materials

The following supporting information can be downloaded at: https://www.mdpi.com/article/10.3390/biology13020114/s1, File S1: Raw biostratigraphic data; File S2: Osteoanatomical measurements, morphometric comparisons and bibliographic references thereof; File S3: Scaled textured photogrammetric models (OBJ, MTL and JPG files).
