# Supplementary figures and images for: Dawn of the Delphinidans: New Remains of Kentriodon from the Lower Miocene of Italy Shed Light on the Early Radiation of the Most Diverse Extant Cetacean Clade
Source: Biology (Basel). 2024 Feb 11;13(2):114. doi: 10.3390/biology13020114 (PMC10887126; doi:10.3390/biology13020114)

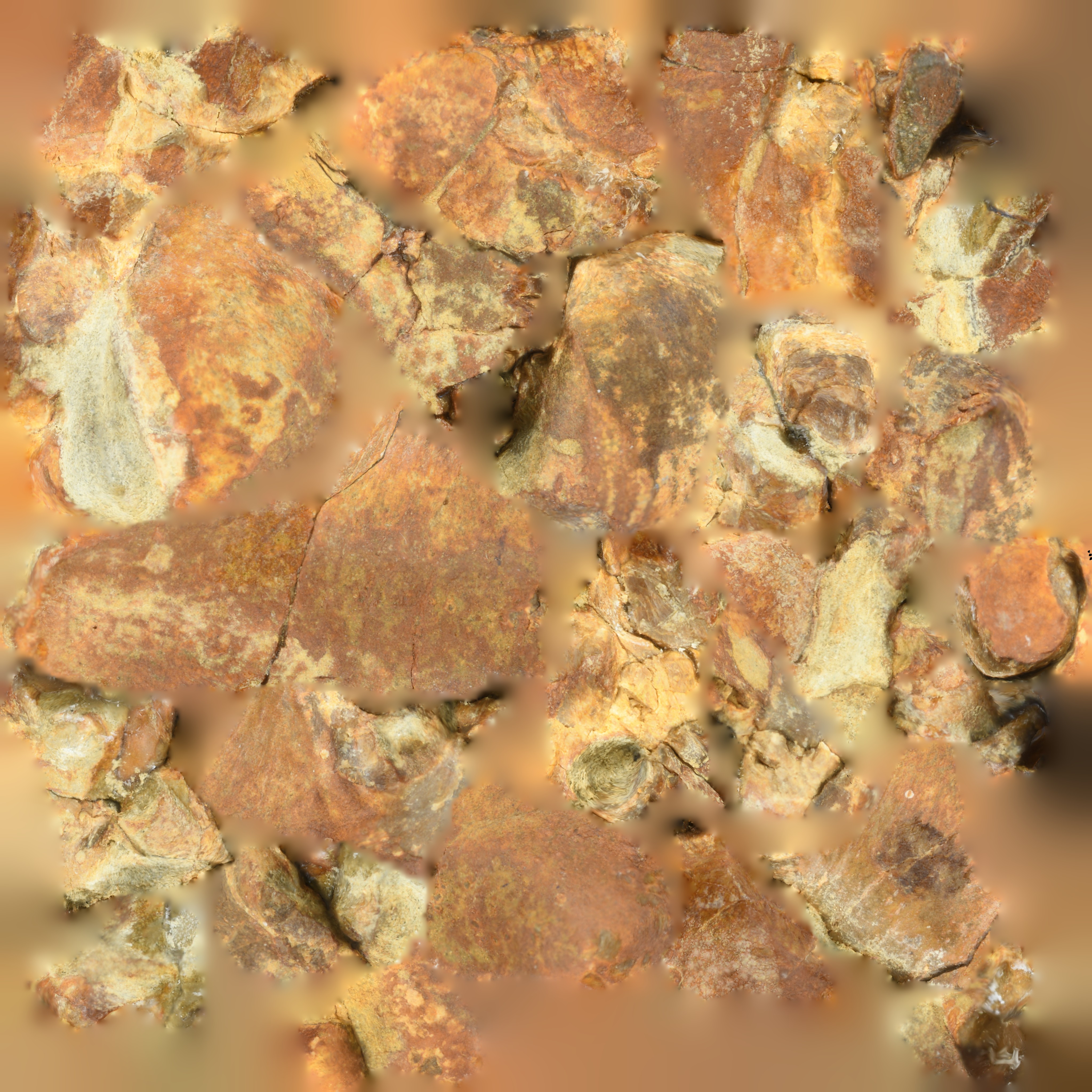

Supplement: Supplementary file 1 [file biology-13-00114-s001.zip › Supplementary File S3/3_Bulla/BullaModel.jpg]

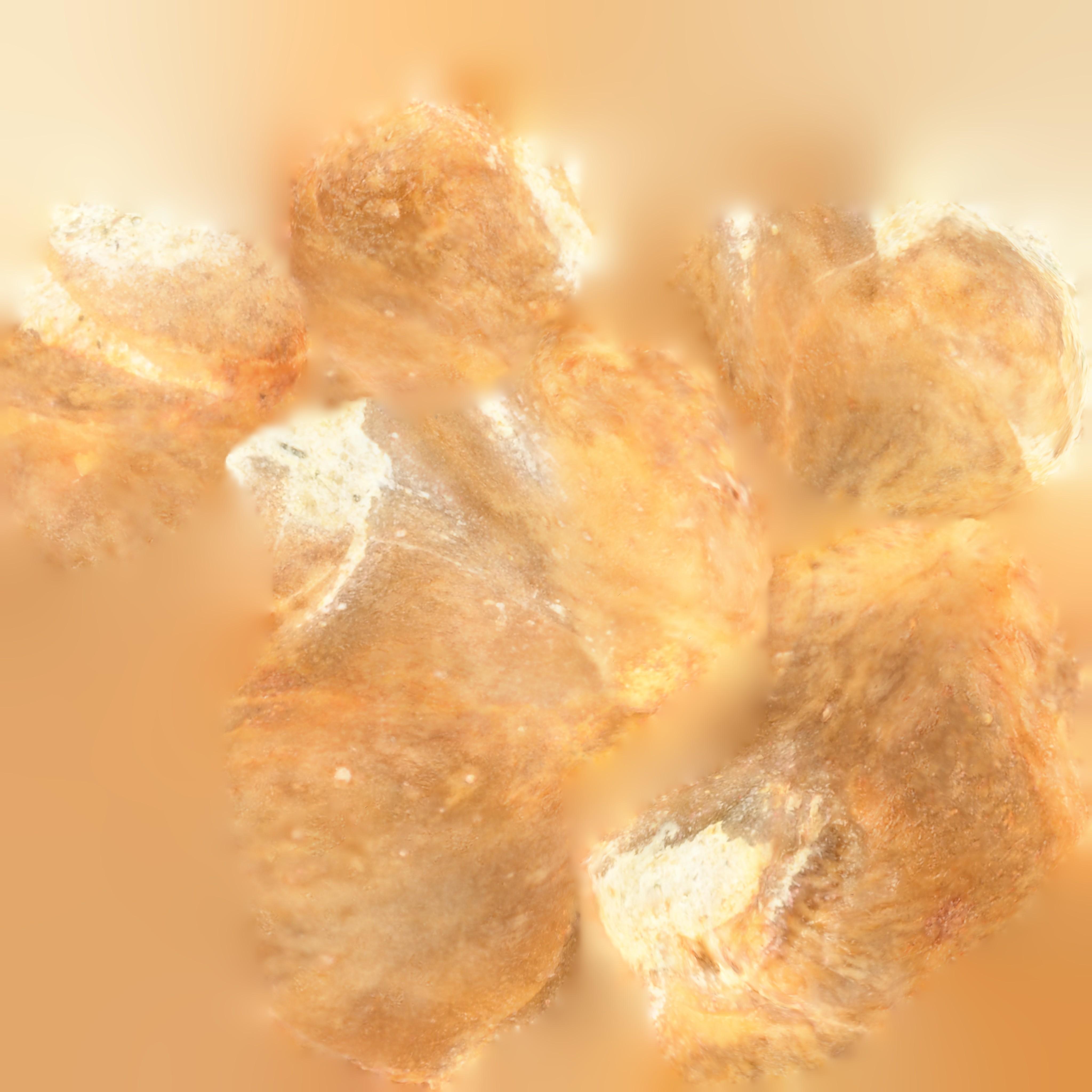

Supplement: Supplementary file 1 [file biology-13-00114-s001.zip › Supplementary File S3/4_Malleus/MalleusModel_S2.jpg]
